# Draft genomes of *Enterococcus faecium* strains isolated from human feces before and after eradication therapy against *Helicobacter pylori*

**DOI:** 10.1016/j.dib.2017.11.069

**Published:** 2017-11-27

**Authors:** Nikita A. Prianichnikov, Maja V. Malakhova, Vlad V. Babenko, Andrei K. Larin, Evgenii I. Olekhnovich, Elizaveta V. Starikova, Dmitry I. Chuvelev, Oksana E. Glushchenko, Andrei E. Samoilov, Alexander I. Manolov, Boris A. Kovarsky, Alexander V. Tyakht, Alexander V. Pavlenko, Elena N. Ilina, Elena S. Kostryukova

**Affiliations:** Federal Research and Clinical Centre of Physical-Chemical Medicine, Malaya Pirogovskaya 1a, Moscow 119435, Russia

## Abstract

The abundance of Enterococci in the human intestinal microbiota environment is usually < 0.1% of the total bacterial fraction. The multiple resistance to antibiotics of the opportunistic *Enterococcus* spp. is alarming for the world medical community because of their high prevalence among clinically significant strains of microorganisms. Enterococci are able to collect different mobile genetic elements and transmit resistance to antibiotics to wide range of Gram-positive and Gram-negative species of microorganisms, including the transmission of vancomycin resistance to methicillin-resistant strains of *Staphylococcus aureus*. The number of infections caused by antibiotics resistant strains of *Enterococcus* spp. is increasing. Here we present a draft genomes of *Enterococcus faecium* strains. These strains were isolated from human feces before and after (1 month) *Helicobacter pylori* eradication therapy. The samples were subject to whole-genome sequencing using Illumina HiSeq. 2500 platform. The data is available at NCBI https://www.ncbi.nlm.nih.gov/bioproject/PRJNA412824.

**Specifications Table**

**Table:** 

Subject area	*Biology*
More specific subject area	*Genomics*
Type of data	*Assembly contigs*
How data was acquired	*The data was acquired on HiSeq. 2500 (Illumina) sequencing platform*
Data format	*Assembly contigs in FASTA format*
Experimental factors	*-//-//-*
Experimental features	*Sequencing was performed according to Illumina sequencing protocols for DNA-seq*
Data source location	*Kazan, Russian Federation*
Data accessibility	Data is available at NCBI repository. https://www.ncbi.nlm.nih.gov/bioproject/PRJNA412824

**Value of the data**

• This data set will be useful for the scientific community, working in the area of medical genomics and/or metagenomics, since it represents the data set of *Enterococcus faecium* genomes, isolated from stool samples of the patients before and after *Helicobacter pylori* eradication therapy.
• This data may be used for comparisons *Enterococcus* spp. genomic data.
• This data will also be valuable for more detailed study of processes occurring in the gut microbiota during the antibiotics administration.

## 1 Data

The following data represents contigs of genomes *Enterococcus faecium* strains isolated from stool samples of two patients before (time point 1) and after (time points 2 and 3) *Helicobacter pylori* eradication therapy. Table 1 contains information about statistics of genomes assembly. In general, on the first time point there are four strains, on the second time point there are two strains and on the third point - one strain.

**Table 1:** Genome assembly statistics of *Enterococcus faecium* strains isolated from patients before and after *Helicobacter pylori* eradication therapy.

**Patient ID**	**Sample ID**	**BioSample ID**	**Time point**	**contigs**	**N50**	**Sum length**	**GC**	**Max length**
HP_003	Hp_5-7	SAMN07728623	1	111	85,683	2,695,017	38.0	165,489
HP_003	Hp_5-10	SAMN07728624	1	77	22,6574	2,840,193	38.3	499,744
HP_003	Hp_6-10	SAMN07728625	2	272	80,066	2,714,038	38.1	261,692
HP_003	Hp_7-8	SAMN07728626	3	175	86,239	2,891,257	38.2	272,078
HP_010	Hp_23-9	SAMN07728628	1	205	45,967	2,688,691	38.0	292,691
HP_010	Hp_23-14	SAMN07728629	1	189	88,668	2,676,966	38.1	191,349
HP_010	Hp_24-3	SAMN07728627	2	232	96,971	2,887,997	37.7	257,098

## 2 Experimental design, materials and methods

### 2.1 Eradication therapy scheme

Eradication therapy was carried out according to the scheme Maastricht 4 [5] (including the antibiotics (clarithromycin, amoxicillin), proton pump inhibitors and bismuth subsalicylate). The data of the cohort assembly and others additional materials may be found in the paper Gluschenko et al. [3].

### 2.2 Isolation

*Enterococcus faecium* strains from two patients (HP_003: three time points and HP_010: two time points) were isolated from stool samples before and after *Helicobacter pylori* eradication therapy. The samples were thawed on ice, 500 mg of sample was then transferred into a new tube and homogenized within 5 sterile phosphate buffered saline. Next, the obtained samples were cultured on dense selective growth media (blood agar, endo agar, simmon's citrate agar and others) in concentration 10-2, 10-4,10-5 ml per one Petri dish. The Petri dishes were put on CO_2_-incubator at a 37 °С until bacterial growing signs appeared. The obtained colonies were re-cultured on growth media for obtaining sufficient biomass and put to the conservation. Next, the identification of species affiliation were performed using Bruker Daltonics MALDI MC Biotyper. The cultures for the isolation of DNA were from the cultivation of isolated single colonies.

### 2.3 DNA extraction

Cell culture was combined with 1.5 ml of Promega Nuclei Lysis Solution buffer and incubated at 70 °С overnight. Silico-zirconium beads (BioSpec Products, USA) with diameters of 0.1 mm (300 mg per sample) and 0.5 mm (100 mg per samples) were added to cell suspension and it was then homogenized by 3 min shaking in MiniBeadBeater (BioSpec Products, USA). Produced mix was incubated at 70 °С for 2 hours. Homogenization and incubations steps were repeated two more times. Further, the DNA was extracted according to Ikryannikova et al. [4].

### 2.4 Sequencing

Genomic DNA libraries were constructed and whole-genome sequencing was performed by HiSeq. 2500 instrument, as described in Gluschenko et al. [3].

### 2.5 Reads preprocessing and assembly

Before genome assembly, quality control was performed by FASTQC [https://www.bioinformatics.babraham.ac.uk/projects/fastqc/] (with default parameters) and Trimmomatic [2] (run keys ILLUMINACLIP:/PATH/TO/ADAPTERS/TruSeq. 3-PE-2.fa:2:30:7:2 TRAILING:25 AVGQUAL:20 MINLEN:50) programs. Genome assembly was made by SPAdes 3.6 [1] in the ‘read error correction and assembling’ mode with --careful key allowing to reduce the number of mismatches and insertions-deletions when assembling the genome of one organism.

## References

[1] Bankevich Anton (2012). SPAdes: a new genome assembly algorithm and its applications to single-cell sequencing. J. Comput. Biol..

[2] Bolger Anthony M., Lohse Marc, Usadel Bjoern (2014). Trimmomatic: a flexible trimmer for Illumina sequence data. Bioinformatics.

[3] Glushchenko Oksana E. (2017). Data on gut metagenomes of the patients with *Helicobacter pylori* infection before and after the antibiotic therapy. Data Brief..

[4] Ikryannikova L.N. (2016). The mystery of the fourth clone: comparative genomic analysis of four non-typeable *Streptococcus pneumoniae* strains with different susceptibilities to optochin. Eur. J. Clin. Microbiol. Infect. Dis..

[5] Malfertheiner Peter (2012). Management of *Helicobacter pylori* infection - the Maastricht IV/Florence consensus report. Gut.

